# Quantitative approaches in electron skin collimation for the practical benefits

**DOI:** 10.1002/acm2.14236

**Published:** 2023-12-05

**Authors:** Dongxu Wang, Jonathan A. Polignani

**Affiliations:** ^1^ Department of Medical Physics Memorial Sloan Kettering Cancer Center New York New York USA

Dear Editor

Methods of electron beam prescribing and treatment planning have evolved. In the past, physicians practiced “the art of electron radiotherapy”: an electron beam treatment would be prescribed based on a physician's clinical training, comfort, and previous experiences. Modern methods apply the concept of “absorbed dose” based on reliable absolute dose calculations and isodose distributions that are displayed in the treatment planning system (TPS). Modern treatment plans are developed to meet target coverage and normal tissue tolerance goals.

Despite advances with modern TPSs, some physicians still prescribe electron plans to a standard isodose line (e.g., 80% or 90% isodose relative to the dose at *d*_max_ along the central axis), and the medical physicist computes the monitor units using tabulated data. Although the physician's comfort level takes precedence and should be respected, this traditional method cannot entirely be reconciled with clinical (in vivo) measurements or the absorbed dose calculations from accurate algorithms. For example, in the presence of a curved patient surface, the dose to clinically relevant areas has been shown to be different than the dose expected from a simple calculation using tabulated data.^1^ Accurate dose computation algorithms can be validated against quantitative dosimetry data. Notably, quantitative dose calculation methods are recommended by the American Association of Physicists in Medicine (AAPM).^2^

Analogously, we ask, “Can we also quantitatively handle custom skin collimation in electron therapy?” Custom skin collimation involves placing custom‐molded lead or lead‐equivalent materials directly on a patient's skin as a tertiary collimation device without being modeled by the TPS. Skin collimation has been used successfully for the past half‐century to protect tissues adjacent to disease sites. This practice predates other popular methods, such as secondary collimation with a Cerrobend field‐shaping insert mounted on the treatment machine.^3^ Skin collimation offers the benefit of a sharp dose profile at the field edge, even for very small field sizes. Typically, the thickness of skin collimation is determined by applying a rule of thumb. The rule of thumb presented in the AAPM's Task Group 25 Report^4^ gives the thickness of lead needed to “stop all electrons” (*t*_lead_) as:

(1) $$t_{\mathrm{lead}}\left(\mathrm{mm}\right)=\frac{E\left(\mathrm{MeV}\right)}{2}$$

Khan^5^ indicates it is “prudent” to add 1 mm, and gives the rule of thumb as:

(2) $$t_{\mathrm{lead}}\left(\mathrm{mm}\right)=1+\frac{E\left(\mathrm{MeV}\right)}{2}$$

These classic rules of thumb, while convenient, are neither quantitative nor practical in many situations. Technical challenges abound for the full quantification of the dosimetric effects of custom skin collimation, but at least the thickness of the collimation can be determined more quantitatively than by using a rule of thumb. TG‐25 recommends that clinicians first determine the desired amount of transmission and then choose the appropriate corresponding thickness to achieve that goal—material transmission data must be measured. TG‐25 states that “in most cases of external shielding, a transmitted dose value of < = 10% is considered acceptable.”^4^ The AAPM advises quantitative approaches in other areas of radiotherapy, such as for analysis of couch tops, beam modifiers, internal electron shielding, and immobilization devices.^6^

Practicality is relevant in custom skin collimation because devices are often fabricated while the patient is waiting in the treatment position. Using less material results in quicker fabrication time. The thickness of the shielding material also affects patient comfort. In the United States, lead sheets can be purchased in thickness increments of 1  or 1.6 mm (1/16^th^ inch). Using the rule‐of‐thumb methods, either 4.5 or 5.5 mm of lead is needed to collimate a 9 MeV beam (Table 2). However, either 5 or 6 mm (in 1 mm thickness increment) or 4.8 or 6.4 mm (in 1.6 mm thickness increment) need to be placed clinically. The resulting device needed to shield a large area is often quite heavy for the patient. Quantitative data show that a 3 mm thickness of lead can achieve < 10% transmission for a 9 MeV beam (Table 1), suggesting that practical shielding for 9 MeV beam would require about 1/3 less lead than what the rule of thumb prescribes (Table 2). This quantitative approach enables a user to make the skin collimation device faster and lighter.

**TABLE 1:** The author's in‐house transmission measurements for different thicknesses of lead or leaded vinyl.

Material and thickness	Transmission for 6 MeV	Transmission for 9 MeV
Lead, 1.0 mm; or Leaded vinyl, 4 mm	55.6%	108.4%^*^
Lead, 1.5 mm; or Leaded vinyl, 6 mm	14.8%	58.5%
Lead, 2.0 mm; or Leaded vinyl, 8 mm	3.4%	26.8%
Lead, 3.0 mm; or Leaded vinyl, 12 mm	2.1%	4.4%

* *Note* that 1 mm lead creates a “bolus” effect for the 9 MeV beam, increasing the surface dose instead of reducing it. The effective point of measurement was 1  or 2 mm below surface, using a Standard Imaging (Middleton, WI) A11 parallel plate chamber and a W2 scintillator. Electron beams were produced by a Varian Truebeam linear accelerator, with a 10 x 10 cm field size, at 100 cm SSD.

**TABLE 2:** Savings using a quantitatively determined lead or leaded vinyl thickness, compared to the rules of thumb.

	Lead			Leaded Vinyl		
	Rule of thumb thickness from TG‐25 or Khan (Equation 1 & 2)	Measured thickness for > 90% reduction	Savings using the quantitatively determined thickness	Rule of thumb equivalent thickness from TG‐25 or Khan (Equation 1 & 2)	Measured thickness for 90% reduction	Savings using the quantitatively determined value
**6 MeV**	3 or 4 mm	2 mm	**1–2 mm or 30%–50%**	12 or 16 mm	8 mm	**4–8 mm or 33%–50%**
**9 MeV**	4.5 or 5.5 mm	3 mm	**1.5‐2.5 mm or 30%–50%**	18 or 22 mm	12 mm	**6–10 mm or 33%–45%**

Another argument for adopting the quantitative approach applies when using lead‐equivalent materials. These alternative materials, such as leaded vinyl, gamma putty, heat‐malleable Matrix, and so forth (Radiation Products Design, Inc., Albertville, MN), offer the practical advantage of being easier to cut and mold than metallic lead sheets. However, a larger thickness of lead‐equivalent materials is required for the same shielding effect as lead. For example, 4 mm thick leaded vinyl is equivalent to 1 mm of lead metal for 6 and 9 MeV electron beams (measured by the author, and described by the vendor).^7^ Using our previous example of a 9 MeV beam, this would result in 18–22 mm thick leaded vinyl (Table 2). Such a thick device does not conform well to skin or clear the limited space under the mounted electron cone at SSD = 100 cm, and it is often too thick to be easily taped onto the skin, especially if it must be placed on a patient's side. If 90% or 95% dose reduction is acceptable by clinicians for the specific case, then the amount of material used can be reduced by about 6–10 mm compared to the “rule‐of‐thumb” thickness (Table 2). If an even lower dose reduction is clinically acceptable, then the material's thickness and weight can be further decreased and would likely be more practical.

Table 1 summarizes the authors’ transmission data for several typical thicknesses of lead or leaded vinyl for 6 and 9 MeV. As the table shows, if the practical limitation is that the clinician wants as much shielding as possible but without a threshold, then lead thickness as low as 1.5 mm can be used for 9 MeV and 1.0 mm can be used for 6 MeV, both achieving more than 40% dose reduction compared to without any lead.

Many electron beam radiotherapy cases could benefit from applying the standard of quantitative analysis instead of rules of thumb to custom skin collimation. The benefits include less time needed to create the device, reduced thickness and weight, and lower costs from using less material. If a department were to adopt the use of lead or lead equivalent materials for custom skin collimation, the medical physicist would relay the measured dosimetric data to the physician, who would indicate if a certain thickness of material yields a clinically acceptable shielding result.

## AUTHOR CONTRIBUTIONS

Dongxu Wang initiated the ideas presented in the document and provided the illustrative data and clinical experience with the materials discussed. Jonathan Polignani provided clinical and literary context, and helped illustrate the relevance of the ideas. Both authors contributed equally to writing the manuscript and both approved the final manuscript.

## CONFLICT OF INTEREST STATEMENT

The authors declare no conflict of interest.
